# Validating Sperm Concentration in Rabbit Cryopreservation Protocol: Implications for Fertility, Litter Size, and Offspring Growth

**DOI:** 10.3390/vetsci12070678

**Published:** 2025-07-18

**Authors:** Michele Di Iorio, Giusy Rusco, Fabrizio Lauriola, Emanuele Antenucci, Alessandra Roncarati, Silvia Cerolini, Michele Schiavitto, Nicolaia Iaffaldano

**Affiliations:** 1 Department of Agricultural, Environmental and Food Sciences, University of Molise, Via De Sanctis snc, 86100 Campobasso, Italy; giusy.rusco@unimol.it (G.R.); f.lauriola2@studenti.unimol.it (F.L.); e.antenucci@studenti.unimol.it (E.A.); nicolaia@unimol.it (N.I.); 2 School of Biosciences and Veterinary Medicine, University of Camerino, Via Circonvallazione 93/95, 62024 Matelica, Italy; alessandra.roncarati@unicam.it; 3 Department of Veterinary Medicine and Animal Science, University of Milan, Via dell’Università 6, 26900 Lodi, Italy; silvia.cerolini@unimi.it; 4 Italian Rabbit Breeders Association (ANCI-AIA), Volturara Appula, 71030 Foggia, Italy; micheleschiavitto@anci-aia.it

**Keywords:** rabbit spermatozoa, cryopreservation protocol, in vivo assessment, fertility, postnatal development

## Abstract

The cryopreservation of rabbit semen is a valuable strategy for genetic resource preservation and efficient artificial insemination, but outcomes remain inconsistent, partly due to variations in sperm concentration per dose. This study aimed to evaluate the in vivo effects of different sperm concentrations (15, 25, 35, 55, and 75 million per straw) on fertility, prolificacy, and offspring growth in nulliparous and multiparous does. A total of 384 rabbit females were inseminated using frozen–thawed semen, and their reproductive performance was compared with fresh semen. Fertility and kindling rates varied with sperm concentration and parity: nulliparous does showed the highest fertility at 15 million sperm/straw (84.4%), while multiparous does reached peak values at 25–55 million/straw (78.1–81.3%). Litter size and live-born kits were consistently higher in multiparous than in nulliparous does. Offspring body weight at 19 and 60 days was influenced by both sperm concentration and maternal parity, with better growth generally observed in multiparous groups. Weaning success remained high across all groups. Our results indicate that sperm concentrations ranging from 15 to 35 × 10^6^/straw are the most suitable for cryopreservation, as they maintain high fertility, prolificacy, and offspring growth, comparable to fresh semen. These results confirm that optimizing sperm concentration during cryopreservation improves reproductive efficiency and that tailoring insemination strategies to the physiological status of the female enhances outcomes. The results provide useful recommendations for improving cryopreservation techniques in rabbit breeding programs.

## Simple Summary

Optimizing reproductive technologies is essential for sustainable rabbit farming and genetic conservation. Cryopreserved semen offers practical advantages, but its use is limited by variable fertility outcomes, partly due to unpredictable sperm concentrations in insemination doses. This study assessed how different concentrations of frozen–thawed sperm affect reproductive performance and offspring growth in nulliparous and multiparous rabbit does. The results demonstrated that low to intermediate concentrations (15–35 million) produced fertility and kindling rates similar to fresh semen, with multiparous females generally achieving higher reproductive performance and heavier offspring. Kit survival and growth were not negatively affected by the use of frozen semen, and optimal weaning rates were observed across all treatments. These findings highlight the importance of adjusting sperm concentration in cryopreservation protocols according to the physiological status of the female, offering practical guidance for enhancing artificial insemination efficiency in rabbit breeding systems.

## 1. Introduction

The cryopreservation of rabbit sperm is a critical tool for enhancing the efficiency of artificial insemination (AI) programs and preserving genetic diversity through semen cryobanks. However, the limited survival rate of rabbit sperm post-cryopreservation poses significant challenges; in fact, the physical and biochemical damage inflicted during the freezing and thawing processes remains a major hurdle. Ice crystal formation, oxidative stress, and membrane destabilization contribute to reduced sperm viability and motility post-thawing [1]. To mitigate these effects, researchers have investigated the role of cryoprotectants, antioxidants, and optimized freezing curves, but their efficacy often varies based on breed-specific responses and donor variability [2,3,4].

Despite advancements in freezing techniques and cryoprotectant formulations over the last decades [1,5,6], the absence of a standardized freezing protocol hampers the consistency of outcomes. One critical aspect contributing to these inconsistencies is the unpredictability of the final sperm concentration in straws, which often fluctuates due to fixed dilution ratios used in freezing protocols [1]. This issue has been highlighted in several studies, including our own works [7,8,9,10,11], as well as in research conducted by other groups [3,12,13,14,15,16,17]. Standardizing sperm concentration is critical to enhancing the efficiency and predictability of rabbit breeding programs. This approach ensures more consistent reproductive performance and supports the broader goal of improving the overall success of rabbit breeding practices.

Recently, to address this issue, we conducted a comprehensive evaluation of five different sperm concentrations in straw (from 15 to 75 million) to determine their effect on post-thaw sperm quality [18]. Our findings demonstrated that mid-range sperm concentrations, specifically between 25 and 35 million sperm per straw, yielded the best in vitro results after thawing. These results suggest that optimizing sperm concentration could be a key factor in enhancing post-thaw performance. However, while these in vitro findings provide valuable insights, their true biological relevance can only be confirmed through in vivo studies, which are essential to assess fertility potential under real reproductive conditions.

In addition to immediate reproductive outcomes, it is crucial to consider the long-term effects of sperm cryopreservation on offspring development. Parameters such as weaning success, body weight at key growth stages, and overall health have not been thoroughly investigated in previous studies. To the best of our knowledge, these aspects have not yet been considered in rabbits. Understanding these aspects is vital for a holistic assessment of cryopreservation protocols, as they directly impact the productivity and sustainability of rabbit breeding programs.

Therefore, in this context, this study aims to evaluate the in vivo effects of different sperm concentrations (15, 25, 35, 55, and 75 × 10^6^ sperm/mL), selected based on our previous study [18], in AI practices, with a comprehensive focus on key reproductive outcomes such as fertility and prolificacy, and subsequent offspring development. By incorporating metrics such as weaning rates and growth performance at 19 and 60 days, we aim to provide a more detailed understanding of how the cryopreservation process and varying sperm concentration influence not only immediate fertility but also long-term productivity.

## 2. Materials and Methods

### 2.1. Animals

A total of 80 adult rabbit bucks (7–9 months of age) and 384 does of the Bianca Italiana breed were used in this study. The does were evenly distributed between nulliparous (n = 192) and multiparous (n = 192) groups. The average age of the multiparous and nulliparous does was 18 and 5.5 months, respectively. Their average body weight was 4.71 kg for the multiparous does and 3.96 kg for the nulliparous does. All animals were sourced from and housed at the Central Breeding Farm of the Italian Rabbit Breeders Association (ANCI-AIA), located in Volturara Appula (Foggia, Italy).

Bucks were housed in WRSA flat-deck cages (46 cm wide × 37 cm high × 60 cm deep). Inseminated does were allocated to two types of cages depending on the reproductive phase, gestation cages (30 cm wide × 40 cm high × 60 cm deep) and farrowing cages (40 cm wide × 47 cm high × 67 cm deep), which were equipped with an additional nest box (40 cm wide × 31 cm high × 24 cm deep).

A photoperiod of 16 h light and 8 h dark was maintained throughout the study period using artificial lighting. All animals were fed a standard pelleted commercial diet for reproductive rabbits, and fresh water was continuously available via nipple drinkers.

### 2.2. Semen Collection, Processing, and Cryopreservation Protocol

The semen doses used in the in vivo trial were previously prepared and described in detail in our earlier publication [18]. Following preparation, the doses were cryopreserved in liquid nitrogen and stored for a period of three months prior to use in this experiment, under standardized conditions to ensure uniformity and preservation of semen quality. For clarity, the key steps of the cryopreservation protocol are outlined below.

Briefly, semen was collected using an artificial vagina; samples were transported to the laboratory at ~30 °C within 30 min, and an aliquot was immediately analyzed for motility, membrane integrity, and concentration. Each semen pool was divided into five aliquots, initially diluted with a tris–citrate–glucose (TCG) extender, cooled to 5 °C, then further diluted with TCG containing 16% DMSO and 0.1 M sucrose to achieve final concentrations of 15, 25, 35, 55, and 75 × 10^6^ sperm/straw. The samples were loaded into 0.25 mL straws, equilibrated, frozen in nitrogen vapor, and stored in liquid nitrogen at −196 °C. Each semen sample was processed and cryopreserved using the freezing technique as outlined by Iaffaldano et al. [7], including the thawing procedure at 50 °C for 10 s.

### 2.3. Comparing the In Vivo Reproductive Performance of Nulliparous and Multiparous Rabbit Does Inseminated with Different Sperm Concentrations per Straw

In this experiment, we evaluated the efficacy of frozen semen doses with varying final sperm concentrations 15, 25, 35, 55, and 75 × 10^6^ spermatozoa per straw in an artificial insemination trial in rabbits. A total of 384 receptive does were used, comprising 192 nulliparous and 192 multiparous animals (31 days postpartum). The does were randomly assigned to twelve treatment groups (n = 32 per group), as detailed in Table 1.

Each doe received a 0.25 mL semen dose according to the assigned treatment. Control groups were inseminated with fresh semen diluted 1:10 with TCG^®^ extender (approximately 35 × 10^6^ spermatozoa), whereas the experimental groups received thawed semen doses at different sperm concentrations.

To synchronize estrus, all does underwent a biostimulation protocol consisting of flushing (increasing feed from 180 g/day to ad libitum three days prior to insemination), cage change (three days prior), and photoperiod extension from 16 to 24 h of light two days before insemination. At the time of insemination, each doe received an intramuscular injection of buserelin acetate (1 μg/doe) to induce ovulation.

Fertility was assessed by abdominal palpation 15 days’ post-insemination, calculated as the number of pregnant does divided by the number of inseminations. At parturition, kindling rate (does giving birth/number inseminated), total kits born per kindling, and kits born alive per kindling were recorded. Postnatal observations included kit weights recorded at 19 and 60 days, as well as the weaning rate at 31 days, expressed as the percentage of kits weaned relative to the number of kits born.

### 2.4. Statistical Analysis

Reproductive performance data (fertility rate, kindling rate, total and live-born kits) and zootechnical parameters (kit weight at 19 and 60 days, weaning percentage) were analyzed using a one-way analysis of variance (ANOVA) to assess the effect of sperm concentration across treatments (fresh semen and frozen semen at 15, 25, 35, 55, and 75 × 10^6^ sperm/straw) within each physiological group (nulliparous and multiparous does). Post hoc comparisons were performed using Duncan’s test, and differences were considered statistically significant at *p* < 0.05.

To compare differences between physiological status (nulliparous vs. multiparous does) within each treatment, an independent-samples *t*-test was applied, with a significance threshold set at *p* < 0.05.

Before analysis, the data were checked for normal distribution using the Shapiro–Wilk test. Continuous data (e.g., litter size, body weight) were expressed as means ± standard error of the mean (SEM), while categorical data (e.g., fertility, kindling, weaning rates) were presented as percentages along with the number of animals per group (n°/total). Statistically significant differences among treatments within the same physiological group were denoted by different superscript letters (a, b, c), while differences between physiological status within the same treatment were indicated using different symbols (*, #). All statistical analyses were conducted using the software package SPSS (IBM SPSS Statistics 23.0 for Windows, 2020; SPSS, Chicago, IL, USA).

## 3. Results

### 3.1. Fertility and Litter Size

The reproductive performance of rabbit does was evaluated after insemination, with Table 2 presenting the fertility and prolificacy rates, as well as the total and live-born kits recorded following artificial insemination using either fresh semen or frozen semen at concentrations of 15, 25, 35, 55, and 75 × 10^6^ sperm per straw.

Regarding fertility, no significant differences were observed between multiparous and nulliparous does when fresh or cryopreserved semen were used. For nulliparous does, fertility rates were 71.9% with fresh semen, increasing to 84.4% with frozen semen at 15 × 10^6^ sperm/straw, but gradually decreasing to 68.8% at 75 × 10^6^ sperm/straw, although the differences were not statistically significant. In multiparous does, fertility with fresh semen was 81.3%. The same value was obtained with frozen semen at a concentration of 55 × 10^6^ sperm/straw, which was significantly higher than the concentrations of 15 and 75 × 10^6^ sperm/straw (*p* < 0.05).

Kindling rates followed a similar trend, as no significant differences were recorded between nulliparous and multiparous does for this parameter, except for frozen semen at a concentration of 15 × 10^6^ sperm/straw, which showed higher values in nulliparous does (*p* < 0.05). In the physiological state of nulliparous does, the significantly higher value of kindling rate was recorded in diluted and frozen semen at a concentration of 15 × 10^6^ sperm/straw compared to concentrations of 35, 55, and 75 × 10^6^ sperm/straw.

In multiparous does, the highest values were recorded with fresh semen (71.9%), with significant differences compared to frozen semen at a concentration of 75 × 10^6^ sperm/straw (43.8%). Meanwhile, the other concentrations showed similar kindling rates, aligning with those of fresh semen.

The physiological status of the does had a more pronounced effect on the total number of kits born and the number of live-born kits. For both parameters, significantly higher values were observed in multiparous does compared to nulliparous ones, both with fresh and frozen semen at various sperm concentrations, except for concentrations of 35 and 75 × 10^6^ sperm/straw.

Litter sizes (total number born) were higher in multiparous does inseminated with fresh semen, averaging 9.6 kits. With frozen semen, the total number of kits born ranged from 6.3 to 7.3 in nulliparous does and from 7.8 to 9.2 in multiparous does across the different sperm concentrations. There were no significant differences in the total number of kits born across treatments. Similarly, the number of live-born kits was higher in multiparous does with fresh semen, averaging 8.9 kits. Frozen semen results varied, with no significant differences in the number of live-born kits across treatments. Nulliparous does had between 5.2 and 6.7 live-born kits, while multiparous does had between 7.7 and 8.9 live-born kits.

### 3.2. Offspring Growth

**Figure 1:**
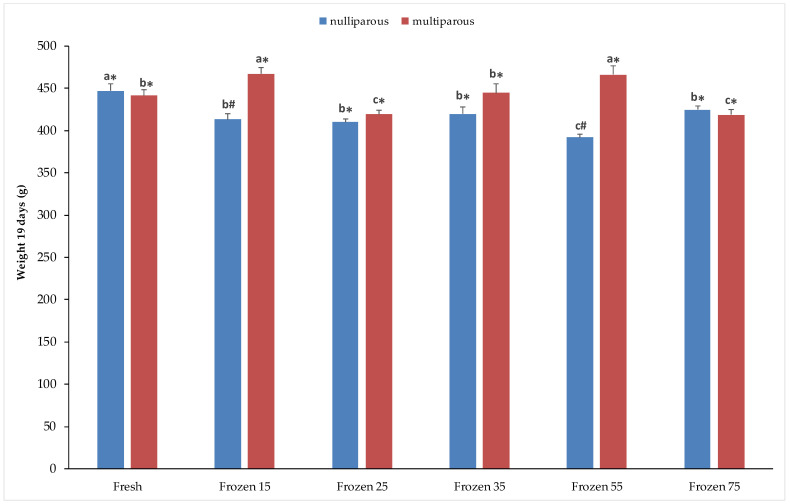
Weight recorded at 19 days of age in kits from nulliparous and multiparous does inseminated with fresh and cryopreserved semen. Different letters (^a–c^) on the bars indicate statistical significance at *p* < 0.05 among fresh and frozen semen at different sperm concentrations/straw, within nulliparous and multiparous does. Different symbols (*^,#^) indicate statistically significant differences at *p* < 0.05 between physiological states (nulliparous and multiparous) in both fresh and frozen semen. Sperm concentrations (15, 25, 35, 55, and 75) are expressed as n° spz × 10^6^/straw.

Figure 1 shows the results obtained for weight at 19 days of age in rabbit kits. In the nulliparous group, the highest weight was recorded in kits born from females inseminated with fresh semen (446.9 ± 8.9 g) compared to frozen semen (*p* < 0.05). The lowest values were obtained at the 55 × 10^6^ concentration (391.8 ± 3.8 g), compared to the other concentrations, which showed similar values among them. Conversely, in the multiparous group, the highest weights were observed with frozen semen at concentrations of 15 and 55, compared to fresh semen and frozen semen at the other sperm concentrations (*p* < 0.05). A significant effect of does’ physiological status was observed with semen cryopreserved at concentrations of 15 and 55 × 10^6^, where significantly higher weights were recorded in multiparous females (467.5 ± 7.3 vs. 412.9 ± 7.4; 466.2 ± 10.7 vs. 391.8 ± 3.8, respectively).

**Figure 2:**
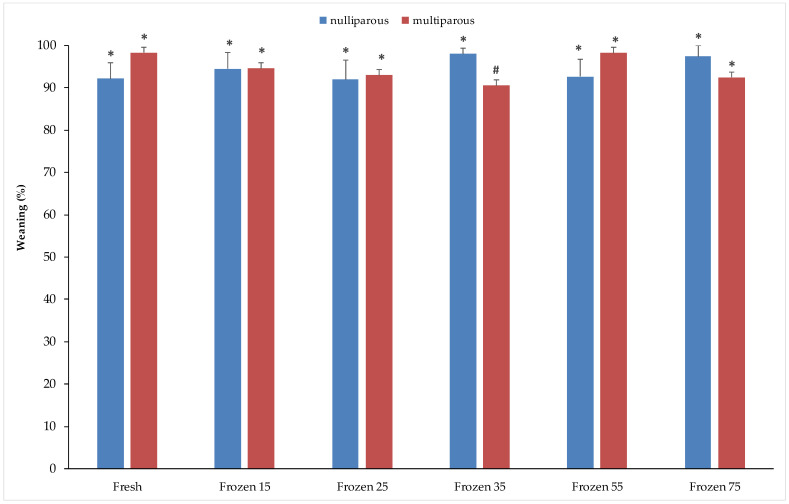
Percentage of weaned kits at 31 days post-partum from nulliparous and multiparous does inseminated with fresh or cryopreserved semen. Different symbols (*^,#^) indicate statistically significant differences at *p* < 0.05 between physiological states (nulliparous and multiparous) in both fresh and frozen semen. Sperm concentrations (15, 25, 35, 55, and 75) are expressed as n° spz × 10^6^/straw.

Figure 2 presents the weaning percentages in nulliparous and multiparous does, using either fresh semen or frozen semen at different concentrations. No significant differences were observed between fresh and frozen semen within each group. However, in the nulliparous group, the highest weaning percentage was recorded with frozen semen at 35 × 10^6^ (98.1 ± 1.4%), whereas in the multiparous group, the highest percentage was obtained with fresh semen (98.3 ± 1.2%). A significant difference between nulliparous and multiparous does was found only when using frozen semen at 35 × 10^6^, with nulliparous females showing a significantly higher weaning percentage than multiparous females (98.1 ± 1.4% vs. 90.6 ± 1.8%).

**Figure 3:**
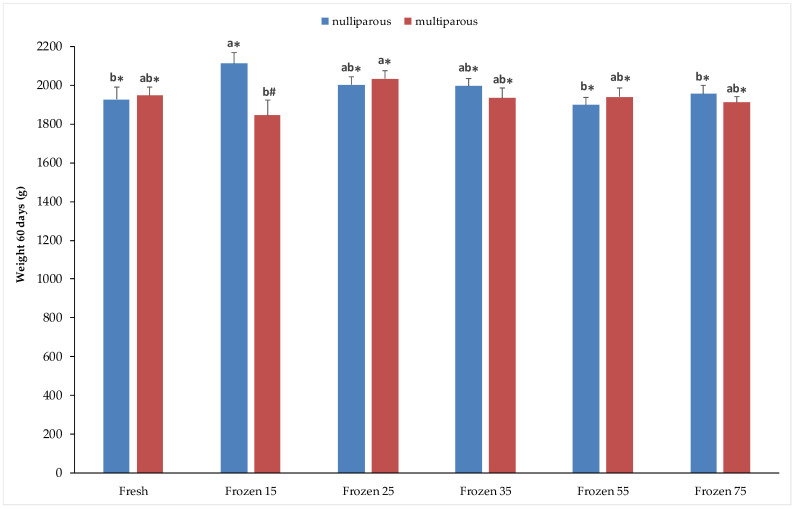
Weight outcomes at 60 days of age in kits from nulliparous and multiparous does inseminated with fresh and cryopreserved semen. Different letters (^a,b^) on the bars indicate statistical significance at *p* < 0.05 among fresh and frozen semen at different sperm concentrations/straw, within nulliparous and multiparous does. Different symbols (*^,#^) indicate statistically significant differences at *p* < 0.05 between physiological states (nulliparous and multiparous) in both fresh and frozen semen. Sperm concentrations (15, 25, 35, 55, and 75) are expressed as n° spz × 10^6^/straw.

The weights recorded 60 days after birth are shown in Figure 3. In the nulliparous group, kits from does inseminated with frozen semen at 15 × 10^6^ concentration showed the highest average weight (2110.6 ± 55.9), significantly higher than those from fresh semen and frozen semen at concentrations of 55 and 75 × 10^6^ (*p* < 0.05). Meanwhile, in the multiparous group, the highest weight was recorded using frozen semen at a concentration of 25 × 10^6^, with significant differences compared to the concentration of 15 × 10^6^. Significant differences between nulliparous and multiparous does were observed only when using frozen semen at a concentration of 15 × 10^6^, with significantly higher values in the nulliparous group compared to the multiparous group (2110.6 ± 55.9 vs. 1845.0 ± 76.9).

## 4. Discussion

The present study was conducted with the aim of validating and confirming, under in vivo conditions, the findings obtained in our previous in vitro investigation, which indicated that intermediate sperm concentrations (25–35 × 10^6^ per straw) were associated with improved post-thaw sperm quality parameters [18], thereby representing the natural progression and in vivo extension of that prior work. Our objective was to expand the understanding of the role that sperm concentration plays not only in determining semen quality but also in influencing fertilizing capacity and the entire reproductive process including key parameters such as fertility, prolificacy, offspring survival, and growth performance up to and beyond weaning.

Specifically, we sought to build a more comprehensive and integrated overview of how sperm concentration used in cryopreservation protocols impacts not only fertilization success but also medium-term productive outcomes, which are often overlooked in the current literature.

### 4.1. Fertility and Kindling Rates

Our findings indicated that sperm concentration is a key determinant of fertility and significantly influences kit body weight, whereas its impact on prolificacy and weaning rate is comparatively limited.

In particular, we observed a trend indicating that fertility and kindling rates were better when frozen semen was diluted to concentrations of 15 and 25 × 10^6^ sperm per straw in nulliparous does, whereas in multiparous does, the highest values were recorded at concentration ranging from 25 to 55 × 10^6^ sperm per straw. A significant decrease in fertility and kindling was observed at higher sperm concentrations of 75 × 10^6^ sperm per straw in both groups of does. This finding is consistent with our previous study, which demonstrated that total and progressive motility were significantly reduced in semen cryopreserved at this concentration [18]. This observation may be explained by the fact that higher sperm concentrations can lead to increased sperm aggregation and decreased motility after thawing, ultimately compromising fertilization success [19]. Furthermore, it is possible that higher sperm concentrations could induce physical damage to sperm cells during the freezing and thawing processes, resulting in a greater loss of sperm viability. Similar trends have been observed in other species, where higher sperm concentrations can result in a reduction in post-thaw motility and fertilization success (ram, [20,21,22]; horse, [23,24]).

The observed decrease in fertility at higher sperm concentrations does not follow a strictly linear pattern, particularly in multiparous does. In this group, fertility peaked within the range of 25–55 × 10^6^ sperm/straw, rather than decreasing steadily. This suggests that the optimal sperm concentration may depend on the physiological status of the female, with multiparous does potentially requiring higher sperm input to achieve maximal fertility outcomes.

Notably, our results indicate that lower and intermediate sperm concentrations may be more favorable for fertility and kindling rates overall. This could be due to reduced sperm competition and the presence of an optimal threshold for successful fertilization [25,26]. Despite these observed trends, physiological status did not significantly affect fertility or kindling rates. This finding is consistent with previous results from our earlier work [7].

### 4.2. Prolificacy

Regarding prolificacy, measured as both total and live-born kits per litter, our findings revealed no significant differences among the different sperm concentrations within each parity group. However, a slight progressive decline in both total and live-born kits was observed at sperm concentrations above 35 × 10^6^/straw. This suggests that, within the tested range (15–75 × 10^6^ sperm/straw), sperm concentration does not critically impact the number of embryos developing to term once fertilization has been successfully achieved.

The lack of prolificacy differences across sperm concentrations may be explained by the biological resilience of early embryonic development, which appears capable of compensating for upstream variations in sperm input. Once oocytes are fertilized, the subsequent stages of cleavage, compaction, blastocyst formation, implantation, and fetal growth are primarily influenced by maternal factors such as uterine environment, hormonal milieu, and immunological tolerance [27,28]. Thus, it can be hypothesized that these processes are relatively independent of the initial sperm concentration used during insemination.

Notably, multiparous does consistently produced larger litters than nulliparous ones. This finding may also be attributed to the extensive reproductive rhythm adopted, whereby multiparous does were inseminated only after the weaning of their previous litter (i.e., 31 days post-partum). Such a management strategy allows for complete physiological recovery and optimization of the reproductive tract prior to next insemination, potentially supporting improved prolificacy outcomes [26,29].

These findings are consistent with previous studies reporting that nulliparous rabbit does generally exhibit higher reproductive efficiency than multiparous females under intensive reproductive rhythms, whereas their performance tends to be lower or comparable under semi-intensive or extensive schedules [29,30,31]. Concordantly, in our study, conducted under an extensive reproductive rhythm, we observed higher prolificacy in multiparous does and comparable fertility rates between nulliparous and multiparous groups.

### 4.3. Offspring Growth and Development

A novel aspect of our study was the assessment of offspring growth and weaning rates. Our findings revealed that kit weight at both 19 and 60 days post-partum was influenced by sperm concentration and also by the parity status of the does. Multiparous females consistently produced heavier kits compared to nulliparous ones at 19 days, which aligns with the well-established concept that experienced breeders offer more favorable gestational and postnatal environments [32,33]. Specifically, within the multiparous group, the use of frozen semen at sperm concentrations of 15 and 55 × 10^6^ sperm/straw was associated with superior offspring growth even compared to fresh semen.

These outcomes suggest that the amount of sperm used during AI may exert subtle but important effects on embryonic development and early postnatal growth during the first 19 days. One possible explanation is the concept of developmental programming, which posits that conditions during early embryonic stages including sperm quality, potential freezing damage, or alterations in sperm DNA can influence fetal growth and metabolic regulation later in life [34]. However, these mechanisms remain largely hypothetical within the context of rabbit reproduction and require further investigation for full understanding.

Sperm cryopreservation can induce molecular alterations such as DNA damage and oxidative stress that, while not preventing fertilization, may impair embryo quality and postnatal development [5,35,36]. Probably, these effects appear more evident in nulliparous does, who have less physiological and maternal maturity compared to multiparous ones. In contrast, multiparous does may better compensate for sperm-related deficits due to improved uterine and maternal conditions. Our results support this, showing that although lower sperm concentrations can support good offspring growth in both groups, the positive effects are more consistent in multiparous.

Interestingly, by 60 days of age, differences in kit weight between nulliparous and multiparous became less pronounced. This may be attributed to the fact that, after weaning, kits grow under similar environmental and nutritional conditions, and the maternal influence, so critical during lactation, is no longer a determining factor [37,38].

Importantly, despite these variations in early growth performance, weaning rates remained uniformly high across all experimental groups, indicating that the variations in growth did not translate into increased mortality of kits during lactation. This is reassuring from both animal welfare and productivity perspectives, suggesting that none of the tested sperm concentrations posed a detrimental risk to neonatal survival.

The importance of assessing offspring development beyond birth is increasingly recognized, as cryopreservation-induced stress may have latent effects on health and productivity [39,40]. Our results provide initial evidence that optimizing sperm concentration during cryopreservation can positively affect not only immediate fertility but also progeny performance, supporting the broader goals of sustainable breeding and genetic resource preservation.

## 5. Conclusions

The findings of this study confirm that sperm concentration used during cryopreservation plays a critical role not only in determining post-thaw semen quality as demonstrated in our previous in vitro research but also in influencing key reproductive and productive outcomes in vivo. Specifically, all measured outcomes including fertility, prolificacy, and offspring growth obtained with frozen–thawed semen in the range of 15 to 35 million/straw were in line with those recorded using fresh semen. This indicates that cryopreserved semen, when used within this concentration window, can effectively substitute fresh semen without compromising reproductive efficiency.

Importantly, this study demonstrates that frozen–thawed semen, when used at appropriate concentrations, is a reliable tool for artificial insemination in rabbits and does not negatively impact the development or growth of progeny. These results provide practical guidance for refining semen cryopreservation protocols and contribute to more efficient, sustainable, and welfare-conscious breeding strategies, with positive implications for both commercial production and genetic conservation.

In addition, the study underscores the importance of considering offspring performance metrics, such as growth and weaning success, when evaluating semen cryopreservation strategies. By extending the analysis beyond fertilization and kindling, this work provides a more holistic view of reproductive efficiency.

## Figures and Tables

**Table 1:** Experimental groups and insemination treatments.

Group	Parity	Semen Type	Sperm Concentration (×10^6^/straw)	Notes
1	Nulliparous	Fresh	~35	Control (TCG^®^ diluted)
2	Multiparous	Fresh	~35	Control (TCG^®^ diluted)
3	Nulliparous	Frozen	15	Thawed semen
4	Nulliparous	Frozen	25	Thawed semen
5	Nulliparous	Frozen	35	Thawed semen
6	Nulliparous	Frozen	55	Thawed semen
7	Nulliparous	Frozen	75	Thawed semen
8	Multiparous	Frozen	15	Thawed semen
9	Multiparous	Frozen	25	Thawed semen
10	Multiparous	Frozen	35	Thawed semen
11	Multiparous	Frozen	55	Thawed semen
12	Multiparous	Frozen	75	Thawed semen

**Table 2:** Reproductive performance obtained in rabbit does after insemination with fresh semen and semen frozen at varying sperm concentration/straws.

Reproductive Performance	Treatment	Doe Status	
		*Nulliparous*	*Multiparous*
Fertility (%) (n°/total)	**Fresh semen**	71.9 ^a^* *(23/32)*	81.3 ^a^* *(26/32)*
	**Frozen semen**		
	**15** **25** **35** **55** **75**	84.4 ^a^* *(27/32)* 81.3 ^a^* *(26/32)* 71.9 ^a^* *(23/32)* 62.5 ^a^* *(20/32)* 68.8 ^a^* *(22/32)*	68.8 ^b^* *(22/32)* 78.1 ^a^* *(25/32)* 78.1 ^a^* *(25/32)* 81.3 ^a^* *(26/32)* 50.0 ^b^* *(16/32)*
Kindling rate (%) (n°/total)	**Fresh semen**	65.6 ^a^* *(21/32)*	71.9 ^a^* *(23/32)*
	**Frozen semen**		
	**15** **25** **35** 55 **75**	81.3 ^a^* *(26/32)* 65.6 ^ab^* *(21/32)* 56.3 ^b^* *(18/32)* 53.1 ^b^* *(17/32)* 56.3 ^b^* *(18/32)*	59.4 ^ab^**^#^** *(19/32)* 56.3 ^ab^* *(18/32)* 68.8 ^ab^* *(22/32)* 68.8 ^ab^* *(22/32)* 43.8 ^b^* *(14/32)*
Total born (mean ± SEM)	**Fresh semen**	7.0 ± 0.4 *	9.6 ± 0.5 **^#^**
	**Frozen semen**		
	**15** **25** **35** **55** **75**	6.3 ± 0.5 * 6.7 ± 0.4 * 7.3 ± 0.3 * 6.6 ± 0.6 * 6.7 ± 0.8 *	9.2 ± 0.6 **^#^** 9.2 ± 0.7 **^#^** 8.4 ± 0.5 * 8.2 ± 0.4 **^#^** 7.8 ± 0.7 *
Live born (mean ± SEM)	**Fresh semen**	6.0 ± 0.5 *	8.9 ± 0.6 **^#^**
	**Frozen semen**		
	**15** **25** **35** **55** **75**	5.2 ± 0.7 * 6.5 ± 0.4 * 6.7 ± 0.5 * 6.2 ± 0.8 * 5.7 ± 0.9 *	8.8 ± 0.6 **^#^** 8.9 ± 0.7 **^#^** 8.0 ± 0.5 * 7.9 ± 0.4 **^#^** 7.7 ± 0.7 *

^a,b^ Different superscript letters within the same column indicate a significant effect of treatment within each doe status (*p* < 0.05), according to one-way ANOVA analysis. *^,**#**^ Different symbols within the same row indicate a significant effect of doe status within each treatment, according to independent *t*-test analysis. Sperm concentrations (15, 25, 35, 55, and 75) are expressed as n° spz × 10^6^/straws.

## Data Availability

Data are contained within this paper.
